# Youth benefit finding and caregiving in a parental illness context: a latent profile analysis

**DOI:** 10.3389/fpsyg.2025.1601162

**Published:** 2025-05-27

**Authors:** Giulia Landi, Kenneth I. Pakenham, Jade Pilato, Géraldine Dorard, Aurélie Untas, Roberto Cattivelli, Silvana Grandi, Eliana Tossani

**Affiliations:** ^1^Department of Psychology, University of Bologna, Bologna, Italy; ^2^Laboratory of Psychosomatics and Clinimetrics, Department of Psychology, University of Bologna, Cesena, Italy; ^3^School of Psychology, The University of Queensland, Brisbane, QLD, Australia; ^4^Laboratoire de Psychopathologie et Processus de Santé, Université Paris Cité, Boulogne-Billancourt, France

**Keywords:** benefit finding, parental illness, youth caregiving, latent profile analysis, health-related quality of life, mental health

## Abstract

**Introduction:**

This study used a person-centred approach to identify patterns of engagement in benefit finding (BF) and caregiving among youth who have a parent with a serious illness.

**Methods:**

A total of 403 youth completed questionnaires in a cross-sectional study.

**Results:**

Latent profile analyses revealed four profiles. The distribution of caregiving and participants across profiles reflected the caregiving continuum. The ‘low BF & caregiving profile’ had the lowest caregiving and the highest proportion of participants at the low end of the continuum, while the ‘moderate BF & extremely high caregiving profile’ had the highest caregiving and the lowest proportion of participants at the high end. The two mid-continuum profiles reflected mid-to-high proportions of caregiving and participants. Results highlighted a corresponding continuum in BF, where engagement varies in sync with caregiving intensity. Profiles differed on demographics, caregiving context, health-related quality of life (HRQoL), and mental health variables. The two mid-continuum profiles reported high caregiving and moderate-to-high BF and evidenced better HRQoL and mental health than the profile at the highest end of the caregiving continuum, but worse HRQoL and mental health than the profile at the lowest end. Despite high caregiving, these two profiles exhibited moderately high HRQoL and mental health, indicating that BF mitigates the adverse impacts of high caregiving. Results also supported the BF theoretical proposal that caregiving must be sufficiently intense to trigger BF.

**Discussion:**

Support services should reduce youth caregiving responsibilities and encourage youth caregivers to explore the positive aspects of their caregiving role.

## 1 Introduction

Youth who have a parent with a serious physical or mental illness are at greater risk of mental health problems compared to their peers with ‘healthy’ parents (Sieh et al., 2010; Cohn et al., 2020; Lacey et al., 2022). Parental illness disrupts family functioning. In particular, the ill parent may be limited in their capacity to fulfil parenting roles and may themselves require physical and psychosocial support due to illness-related limitations, which necessitates a redistribution of roles (Pedersen and Revenson, 2005; Pakenham and Cox, 2012). In this context, children often assume additional caregiving roles, some of which are demanding and typical of those undertaken by adults (Hendricks et al., 2021; Landi et al., 2022d). Concerningly, intensive youth caregiving may interrupt normative development and pose a threat to physical and psychosocial functioning. Indeed, higher caregiving levels are related to poorer mental health and psychosocial outcomes in youth (Pakenham and Cox, 2015; Landi et al., 2022b). Specifically, the mental health of youth aged 11 to 24 years has recently been identified as a global public health priority due to the significant biopsychosocial challenges faced during their transition from adolescence to young adulthood (McGorry et al., 2024). This age range has been used in prior and ongoing youth caregiving research (e.g., Pakenham et al., 2006; Metzing et al., 2020; Landi et al., 2025b), and was therefore chosen as the focus of the present study.

Research on youth caregiving has primarily focused on risk factors, often overlooking protective factors such as benefit finding (BF), which involves identifying positives in stressful situations like caregiving. Although scarcely researched in the youth caregiver field, BF is associated with better mental health in adult caregivers (Pakenham, 2005; Cassidy, 2013) and in youth within the broader associated literature (Kritikos et al., 2021; von Rezori et al., 2024). Hence, this study investigates the interplay between BF and caregiving in youth with a seriously ill parent. Using a person-centred approach, we analyse variations in levels of BF and caregiving to identify subgroups of youth who exhibit similar patterns of engagement in these variables.

### 1.1 Youth caregiving

Youth caregiving has been conceptualized as a continuum, ranging from minor caregiving activities like household chores to regularly engaging in higher amounts of caregiving activities typically performed by adults. Youth at the higher end of this continuum who provide regular and substantial care to ill or disabled family members are referred to as ‘young carers’, particularly in the context of identifying those who require welfare support (Becker, 2007; Joseph et al., 2020). However, given the unique circumstances of undertaking any type or level of caregiving in the context of a seriously ill family member, ‘young carer’ has also been used more inclusively, encompassing all youth along the caregiving continuum caring for an ill or disabled family member, particularly a parent (e.g., Newman, 2002; Pakenham et al., 2006).

A wide range of caregiving activities are undertaken by youth who have a parent with a serious illness, including providing emotional support, undertaking household chores, managing finances, and rendering personal care (Nagl-Cupal et al., 2014; Metzing et al., 2020; Untas et al., 2022). However, an important dimension of caregiving activities is the youth’s experience of the impacts on intra-personal, family, and social areas. A widely used youth caregiving instrument that comprehensively measures these dimensions is the Young Carer of Parents Inventory-Revised (YCOPI-R) (Cox and Pakenham, 2014; Landi et al., 2022a). The YCOPI-R assesses the sense of responsibility associated with key caregiving activities and related experiences across the caregiving continuum. In the present study, youth caregiving is operationalized by the YCOPI-R.

Research has predominantly highlighted the negative impacts of youth caregiving on mental and physical health as well as psychosocial domains (e.g., leisure, employment, education, and socialization). In the context of parental illness, greater engagement in youth caregiving has been identified as a strong predictor of poorer mental health and psychosocial outcomes (Cox and Pakenham, 2014; Pakenham and Cox, 2015). However, qualitative research has also identified positive impacts of caregiving on youth, including enhanced self-esteem (Bolas et al., 2007; Metzing-Blau and Schnepp, 2008), increased maturity (Metzing-Blau and Schnepp, 2008; McDougall et al., 2018), closer family ties (Earley et al., 2007; McDonald et al., 2009; Nichols et al., 2013), and skills development along with a sense of achievement (Bolas et al., 2007). These findings have sparked an interest in the extent to which youth caregivers engage in BF.

### 1.2 Benefit finding

BF refers to the process of identifying benefits in adversity (Tennen and Affleck, 2002). BF is typically conceptualized as a meaning-making process involving finding positives in adversity, which helps to restore meaning that has been disrupted by hardship (Park and Folkman, 1997; Janoff-Bulman and Yopyk, 2004; Tedeschi and Calhoun, 2004). According to this perspective, significant negative life events, such as illness in a loved one and the associated caregiving, can disrupt fundamental assumptions about the world and the self, triggering a state of meaninglessness along with associated distress. Finding benefits in adversity involves re-evaluating the adverse circumstances positively, thereby mitigating the negative implications and protecting self-worth (Taylor, 1983). This process fosters new meanings and assists in integrating the adversity into a personalized life perspective (Janoff-Bulman and Yopyk, 2004).

The few studies that have investigated BF in youth caregivers show that in various caregiving contexts, youth have reported benefits or gains from their caregiving, including personal growth, strengthening of relationships, and changes in priorities and goals (Pakenham et al., 2007; Pakenham and Cox, 2018; Areguy et al., 2019). Mounting evidence suggests that BF in youth caregiving mitigates the adverse effects of caregiving on mental health (Wepf et al., 2022). Higher BF has been associated with better mental health and psychosocial outcomes in youth caregivers (Pakenham and Bursnall, 2006; Pakenham et al., 2007; Cassidy and Giles, 2013; Wepf et al., 2022).

BF theory proposes that the stressful situation or adversity needs to be of sufficient intensity to disrupt existing meaning structures and, hence, trigger a search for new meanings, including searching for positives in adversity (Janoff-Bulman and Yopyk, 2004; Tedeschi and Calhoun, 2004). Consistent with this proposal, higher youth caregiving engagement is likely to trigger a search for meaning in the caregiving role and thereby evoke BF. Supporting this proposal, higher levels of caregiving are associated with greater BF in adult (e.g., Pakenham, 2005; Pakenham and Cox, 2008), and youth (e.g., Cassidy and Giles, 2013) caregivers. Furthermore, one study showed that while increased youth caregiving in a parental illness context had a detrimental effect on youth psychosocial outcomes, these effects were ameliorated by increased BF associated with caregiving, which in turn improved mental health and psychosocial outcomes (Pakenham and Cox, 2018).

Most BF and youth caregiving studies have adopted a variable-oriented approach (Lanza and Cooper, 2016), focusing on average levels of BF and caregiving, which obscures distinct patterns of variation in the relationship between the two variables. Given qualitative data attesting to the variability in these variables among individuals due to variations in personal characteristics and caregiving contexts (Gough and Gulliford, 2020; Jamir Singh et al., 2023), a person-oriented approach would better capture individual differences in BF and caregiving. This approach considers individuals as dynamic systems (Bergman and Andersson, 2010), facilitating a comprehensive analysis of how individuals vary in their engagement in BF and caregiving. By identifying subgroups of youth caregivers with similar patterns of engagement in both variables, this method can unravel the complex interplay between BF and caregiving and their effects on mental health. To our knowledge, no published studies have employed this approach to examine patterns of covariation in BF and caregiving among youth or adult caregivers.

### 1.3 The present study

This study investigates variations in the interplay between BF and caregiving in youth who have a parent with a serious physical or mental illness. We adopt a person-centred methodology using latent profile analysis (LPA) to categorize youth caregivers into subgroups based on their distinctive BF and caregiving profiles. We also investigate socio-demographic and caregiving context variables characterizing each subgroup and, relevant to global public health concerns about youth mental health, we identify those subgroups at risk for mental health problems. Thus, this study has three objectives:

Objective 1: to delineate empirically distinct profiles of BF and caregiving in youth caregivers in a parental illness context. Given that LPA is inherently exploratory and in the absence of theoretical and empirical data suggesting patterns of BF and youth caregiving that might emerge, we pose no hypotheses regarding the number or nature of these profiles.

Objective 2: to explore associations between membership in the profiles and socio-demographic and caregiving context variables.

Objective 3: to investigate differences across the profiles in health-related quality of life (HRQoL) and mental health (internalizing and externalizing behaviors) after controlling for the effects of relevant socio-demographic and caregiving context variables.

## 2 Materials and methods

### 2.1 Participants and procedures

A total of 403 youth with a parent who had a serious physical or mental health condition participated in this cross-sectional survey study. Recruitment was conducted in Italy through a convenience sampling method via the dissemination of study information in educational institutions, local community illness organizations, healthcare facility waiting rooms, and various social media. Inclusion criteria included fluency in Italian, currently living with a parent who has a serious illness, and being aged 11–24 years. The exclusion criterion was the presence of severe medical conditions in the participant or other family members besides the parents. Youth interested in study participation contacted the research team. A research team member then administered the paper-based questionnaire during an in-person meeting, typically conducted at the participant’s home. For participants under the age of 18, informed consent was obtained from both parents, while participants aged 18 years or older provided consent themselves. This procedure facilitated participant cooperation and understanding of the study, enhancing the quality and completeness of data collection. No *a priori* power analysis was conducted. Adequacy of the sample size was based on recruitment feasibility and consideration of sample sizes in previous LPA studies in the youth caregiving and mental health field, which typically ranged from approximately 200 to 500 participants (e.g., Kircanski et al., 2017; Nylund-Gibson and Choi, 2018; Bonadio et al., 2022). The obtained total sample of 403 participants was deemed sufficient to conduct reliable latent profile modelling and subgroup comparisons.

### 2.2 Measures

#### 2.2.1 Socio-demographics and caregiving context variables

Participants provided information on gender, age, current studying or working status (yes/no), and socio-economic status (SES). The latter was assessed using the Family Affluence Scale-II (Boyce et al., 2006), a four-item measure of family material wealth. Scores are summed, with higher scores indicating greater family wealth (categorized into three affluence levels: 0–2 = low, 3–5 = medium, 6–9 = high) (Boyce et al., 2006). Participants also provided information on caregiving context variables: number of family members, which parent had an illness (mother, father, or both), and the parent’s type of illness (physical or mental). Finally, youth caregivers rated the level of caregiving (“help”) they provided to the ill parent on a five-point scale (1 = *none* to 5 = *lots*).

#### 2.2.2 Benefit finding

BF was assessed with the Italian version of the 18-item Young Carer Benefit Finding Scale (Pakenham and Bursnall, 2006; Pakenham et al., 2007; Pakenham and Cox, 2018). Items reflect a range of BF themes associated with youth caregiving, including caregiving gains, personal growth, strengthening of relationships, appreciation of life, health gains, spiritual growth and positive changes in life priorities. Items are rated on a 5-point scale (0 = *strongly disagree* to 4 = *strongly agree*). Items were averaged with higher scores reflecting higher caregiving BF (range 0–4). In the absence of a validated Italian version of the scale, the measure was translated into Italian following standard cross-cultural adaptation guidelines (van Widenfelt et al., 2005). Consistent with these recommendations, we undertook the following: independent forward translation by two bilingual experts, reconciliation of discrepancies, back-translation into English by an independent translator, and review by a committee of experts to ensure conceptual, cultural, and linguistic equivalence. A confirmatory factor analysis was conducted. Fit indices were satisfactory for the original one-factor model: *χ*^2^ (368) = 237.225, *p* < 0.001; CFI = 0.917; TLI = 0.903; RMSEA = 0.059, 90% CI = 0.047, 0.071. The observed McDonald’s Omega was 0.91.

#### 2.2.3 Youth caregiving

The validated Italian version (Landi et al., 2022a) of the Young Carer of Parents Inventory-Revised (YCOPI-R) (Cox and Pakenham, 2014) was employed to assess youth caregiving. This 39-item questionnaire consists of eleven subscales: caregiving responsibilities, activity restriction global, activity restriction study/work, isolation, perceived maturity, worry about parents, caregiving stigma, caregiving confidence, caregiving resentment, caregiving guilt, and caregiving information. Items are rated on a 5-point scale (0 = *strongly disagree* to 4 = *strongly agree*). Scores were averaged and higher scores on each dimension indicate greater caregiving engagement (range for each dimension: 0–4). The observed McDonald’s Omegas ranged were 0.70 to 0.85.

#### 2.2.4 Health-related quality of life

The Italian validated version of the Kidscreen-27 (Ravens-Sieberer et al., 2007) is a 27-item questionnaire that evaluates HRQoL in youth. Responses are rated on a 5-point scale (0 = not at all/never to 4 = extremely/always). The overall HRQoL score is derived by summing the responses, with higher scores reflecting better HRQoL (range 27–135). The observed McDonald’s Omega was 0.92.

#### 2.2.5 Mental health

Youth mental health was assessed using the Italian version (Frigerio et al., 2004) of the Youth Self-Report (Achenbach and Rescorla, 2001). This scale is a widely used standardized measure of youth internalizing and externalizing behaviors. Items are rated on a 3-point scale (0 = not true to 2 = very true). Items are summed, with higher scores indicating more problem behaviors (internalizing range: 0–64 and externalizing range: 0–70). The observed McDonald’s Omegas were internalizing 0.90, externalizing 0.85.

### 2.3 Data analysis approach

To meet the first study objective, LPAs were applied to the observed values of BF and caregiving, using *Mplus 8.3* with the robust maximum likelihood estimator (Muthén and Muthén, 2018). The dataset exhibited a minimal missing value rate of 0.74%. The Little’s MCAR test, which adjusts for large sample size sensitivity via the χ^2^/*df* ratio (Little, 1988; Bollen, 1989), confirmed the randomness of missing data. Consequently, the Full Information Maximum Likelihood method was adopted to manage missing data.

LPA involves a form of mixed modelling, which probabilistically groups participants into profiles that exhibit significant similarities across variables (Berlin et al., 2014). Optimal model selection is based on several criteria: a lower Sample Size Adjusted Bayesian Information Criterion (SSA-BIC) indicative of better models; entropy above 0.75 indicating accurate classification; the adjusted Lo–Mendell–Rubin Likelihood Ratio Test, where a non-significant result suggests that adding profiles does not significantly enhance the model; and the interpretability and theoretical justification of each profile. Importantly, profiles must represent at least 5% of the sample for meaningful interpretations.

All other analyses were conducted using SPSS version 24. The second research objective was explored through multinomial logistic regressions, investigating associations between socio-demographics and caregiving context variables (independent variables) and profile memberships (dependent variable). To address the third objective, univariate ANCOVAs assessed differences in HRQoL and mental health (internalizing and externalizing behaviors) across profiles, controlling for the effects of socio-demographic and caregiving context variables that significantly differed in the multinomial logistic regressions. Cohen’s d was used to report effect sizes—large = 0.80, moderate = 0.50, and small = 0.20 (Ellis, 2010). Significant ANOVAs were followed by *post-hoc* Tukey HSD.

## 3 Results

### 3.1 Sample characteristics

Of the 403 youth participants with a seriously ill parent, 59.55% were female, with an average age of 17.70 years (SD = 3.65). Socio-demographics and caregiving context variables are reported in Table 1.

**Table 1:** Sample characteristics (*N* = 403).

Variable	% (*n*)	*M* (SD)	Range
Socio-demographics			
Gender: female	59.55 (240)		
Age, years		17.70 (3.65)	11–24
Currently study	83.87 (338)		
Currently working	29.03 (117)		
SES		5.68 (1.73)	1–9
Caregiving context variables			
Number of family members		4.03 (1.15)	1–8
Ill mother	63.28 (255)		
Ill father	30.02 (121)		
Both parents ill	6.70 (27)		
Parental physical illness^a^	80.65 (325)		
Parental mental illness^b^	19.35 (78)		
Amount of caregiving		2.95 (0.83)	1–5

a Including cancer (38.15%), diabetes (19.07%), nervous system diseases (14.77%), rheumatic diseases (8.00%), digestive diseases (3.69%), autoimmune diseases (3.08%), circulatory system diseases (3.38%), musculoskeletal-related diseases (2.48%), respiratory diseases (2.46%), physical and sensorial disabilities (2.15%), genitourinary diseases (1.54%), and infectious diseases (1.23%).

b Including substance use disorders (61.91%) and anxiety, depression, or obsessive-compulsive disorders (38.09%).

### 3.2 Latent profile analysis of BF and youth caregiving

**Figure 1:**
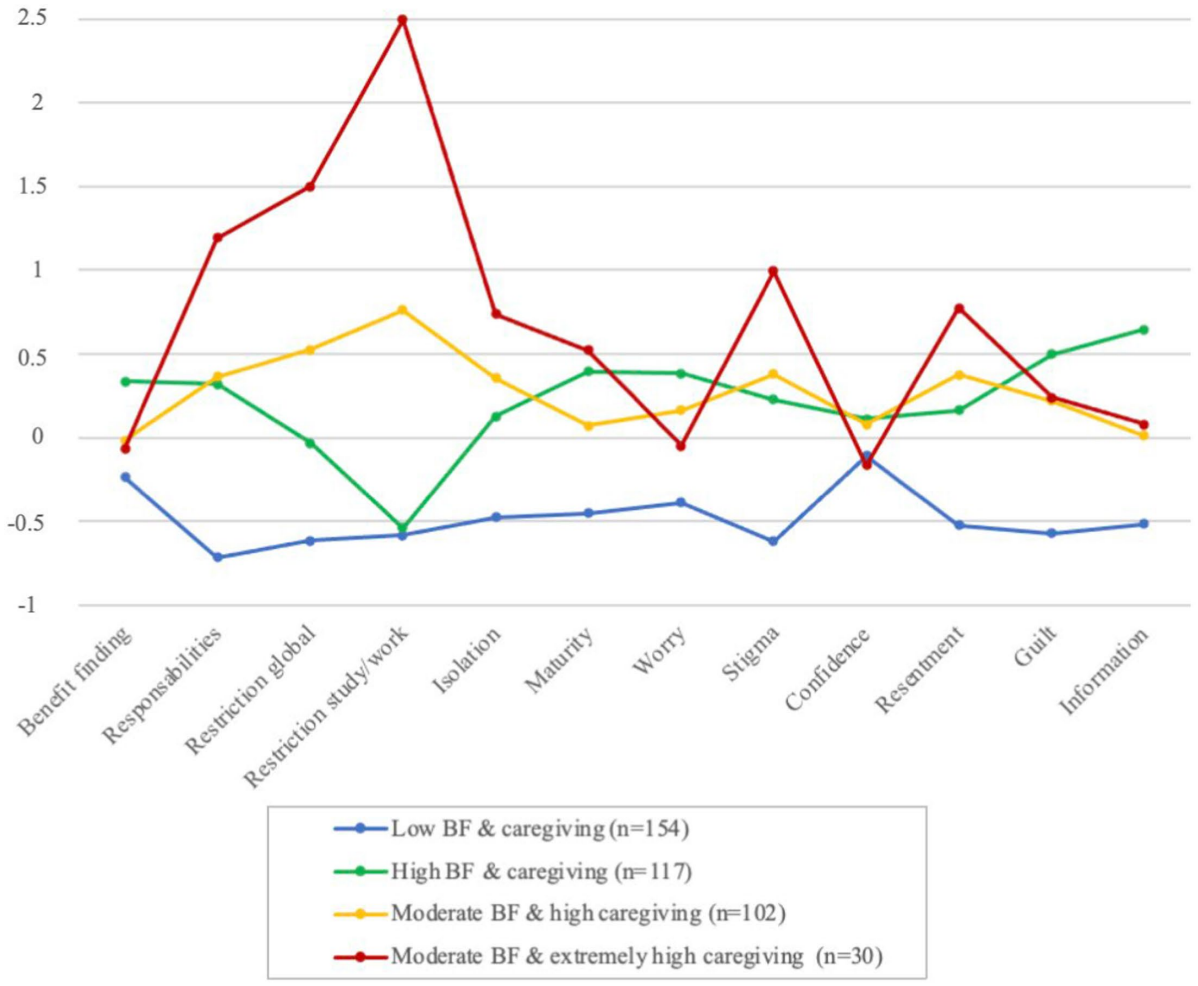
Latent profiles of BF and caregiving based on the total sample *Z* scores (*N* = 403).

LPAs were conducted on the observed values of BF and caregiving, exploring one to five profiles. Fit indices are presented in Table 2. The four-profile model was the most efficient in terms of parsimony, showing lower SSA BIC values than the two- and three-profile models. Adding a fifth profile increased SSA BIC and identified a group representing only 2.48% of the sample, reducing its interpretability. Consequently, the four-profile model was selected for its satisfactory entropy (0.796), reflecting good classification accuracy. Table 3 presents means and standard deviations for BF and caregiving for each profile, along with significant differences among the profiles. Figure 1 depicts *z* scores for BF and caregiving across profiles relative to the total sample.

**Table 2:** Latent profile analyses of BF and caregiving.

Classes specified	SSA BIC	Entropy	Adj. LMR-LRT	Group prevalence %				
				1	2	3	4	5
1	7260.496	–	–	100				
2	6989.284	0.896	312.689*	59.06	40.94			
3	6892.964	0.750	141.366*	47.15	39.95	12.90		
**4**	**6836.448**	**0.796**	**102.374**	38.21	29.03	25.31	7.45	
5	12053.245	0.796	52.950	42.43	33.00	16.13	5.96	2.48

^∗^*p* < 0.05, **p < 0.01, ***p < 0.001. SSA BIC=Sample Size Adjusted Bayesian Information Criterium; Adj. LMR-LRT, adjusted Lo–Mendell–Rubin likelihood ration test. Bold indicates the best-fitting solution (*N* = 403).

**Table 3:** Means and standard deviations of BF and caregiving for each profile.

Variable	Total sample (*N* = 403)	Low BF & caregiving (*n* = 154)	High BF & caregiving (*n* = 117)	Moderate BF & high caregiving (*n* = 102)	Moderate BF & extremely high caregiving (*n* = 30)	Test of group difference	Cohen’s *d* for largest observed difference in pairs among profiles
	*M* (SD)	*M* (SD)	*M* (SD)	*M* (SD)	*M* (SD)		
BF	2.17 (0.94)	1.95 (0.90)^a,b^	2.49 (0.85)^a,b^	2.16 (0.93)	2.11 (1.21)	*F* (3,403) = 6.97**	0.48
Caregiving responsibilities	1.51 (0.79)	0.95 (0.58)^a,b,c,d^	1.77 (0.74)^a,b,d^	1.80 (0.58)^a,c,d^	2.46 (0.57)^a,b,c,d^	*F* (3,403) = 76.76**	1.52
Caregiving experiences							
Activity restriction global	1.07 (0.89)	0.53 (0.65)^a,b,c,d^	1.05 (0.73)^a,b,c,d^	1.54 (0.67)^a,b,c,d^	2.41 (0.92)^a,b,c,d^	*F* (3,403) = 8318**	1.58
Activity restriction study/work	0.54 (0.72)	0.12 (0.26)^a,c,d^	0.15 (0.23)^b,c,d^	1.10 (0.32)^a,b,c,d^	2.35 (0.57)^a,b,c,d^	*F* (3,403) = 636.69**	4.38
Isolation	1.75 (1.07)	1.24 (1.01)^a,b,c,d^	1.89 (1.08)^a,b,d^	2.13 (0.85)^a,c^	2.54 (0.88)^a,b,d^	*F* (3,403) = 26.01**	0.88
Caregiving maturity	2.45 (0.93)	2.03 (0.97)^a,b,c,d^	2.82 (0.83)^a,b,c^	2.51 (0.73)^a,b,c^	2.93 (0.84)^a,d^	*F* (3,403) = 22.59**	0.82
Worry about parents	2.90 (0.85)	2.57 (0.90)^a,b,c^	3.22 (0.73)^a,b^	3.04 (0.71)^a,c^	2.86 (0.88)	*F* (3,403) = 15.96**	0.69
Caregiving stigma	1.25 (0.87)	0.71 (0.62)^a,b,c,d^	1.44 (0.90)^a,b,d^	1.57 (0.71)^a,c,d^	2.11 (0.76)^a,b,c,d^	*F* (3,403) = 49.63**	1.22
Caregiving confidence	1.89 (0.78)	1.81 (0.79)	1.98 (0.80)	1.96 (0.70)	1.77 (0.93)	*F* (3,403) = 1.56	0.22
Caregiving resentment	0.90 (0.79)	0.49 (0.57)^a,b,c,d^	1.03 (0.77)^a,b,d^	1.20 (0.76)^a,c^	1.51 (0.92)^a,b,d^	*F* (3,403) = 31.83**	0.98
Caregiving guilt	1.43 (0.90)	0.92 (0.74)^a,b,c,d^	1.88 (0.83)^a,b^	1.63 (0.85)^a,c^	1.64 (0.81)^a,d^	*F* (3,403) = 35.96**	1.04
Caregiving information	2.07 (1.17)	1.47 (1.11)^a,b,c,d^	2.83 (0.94)^a,b,c,d^	2.09 (1.08)^a,b,c^	2.17 (0.86)^a,b,d^	*F* (3,403) = 38.29**	1.07

^∗^*p* < 0.01, **p < 0.001. Means sharing the same superscript notation (a,b,c,d) were found to differ significantly in the post-hoc comparisons. Cohen’s d was derived from the eta-squared values obtained from the ANOVAs.

The first profile, encompassing 38.21% (*n* = 154) of participants, featured low BF and caregiving and was identified as the “low BF & caregiving profile.” The second profile, representing 29.03% (*n* = 117) of participants, exhibited elevated BF and caregiving. Most caregiving dimension scores were above their corresponding sample means, except for the two activity restriction dimensions, which were below the sample mean. This profile was identified as the “high BF & caregiving profile.” The third profile, accounting for 25.31% (*n* = 102) of the sample, featured moderate BF and elevated caregiving across all dimensions and was named the “moderate BF & high caregiving profile.” The fourth profile included 7.45% (*n* = 30) of the sample and exhibited moderate BF and extremely high caregiving with exceedingly high caregiving responsibilities and very high caregiving on most of the other dimensions. This profile was labelled the “moderate BF & extremely high caregiving profile.”

Only two profiles differed on BF, with the ‘low BF & caregiving profile’ having a significantly lower mean than the ‘high BF & caregiving profile’. Regarding caregiving, notable differences emerged. All profiles significantly differed in caregiving responsibilities except the ‘high BF & caregiving profile’ and the ‘moderate BF & high caregiving profile’, which exhibited similar levels. The ‘moderate BF & extremely high caregiving profile’ recorded extremely high caregiving responsibilities and high levels in the other caregiving dimensions, significantly exceeding those of the ‘low BF & caregiving profile’, which registered the lowest levels. Differences in three caregiving dimensions—activity restriction global, activity restriction study/work, and caregiving information—were noted between the ‘high BF & caregiving profile’ and the ‘moderate BF & high caregiving profile’. Specifically, compared to the ‘moderate BF & high caregiving profile’, the ‘high BF & caregiving profile’ had significantly lower means across all these caregiving dimensions.

### 3.3 Differences in socio-demographics and caregiving context variables across profiles

Regarding objective 2, multinomial logistic regressions examined associations between profile membership and socio-demographic and caregiving context variables (see Table 4). The analyses modelled the odds of belonging to each profile against a reference profile (‘high BF & caregiving profile’). Among the socio-demographics, age and SES significantly distinguished profile classification. Specifically, SES predicted the ‘low BF & caregiving profile’ when contrasted with the ‘high BF & caregiving profile’ (B = 0.25, SE = 0.08, *p* < 0.001), indicating each SES unit increase made classification in the ‘low BF & caregiving profile’ 1.28 times more likely (95% CI = 1.09–1.50). Age also distinguished between the ‘moderate BF & extremely high caregiving’ and the ‘high BF & caregiving’ profiles (B = 0.15, SE = 0.08, *p* < 0.05), with each additional year increasing the odds of being classified in the ‘moderate BF & extremely high caregiving profile’ by a factor of 1.17 (95% CI = 1.00–1.36).

**Table 4:** Multinomial logistic regression of the link between demographics and caregiving context variables and profile classification.

Variable	Low BF & caregiving (*n* = 154)		Moderate BF & high caregiving (*n* = 102)		Moderate BF & extremely high caregiving (*n* = 30)	
	*B* (*SE*)	Odds ratio (95% CI)	*B* (*SE*)	Odds ratio (95% CI)	*B* (*SE*)	Odds ratio (95% CI)
Demographics						
Gender (female = 0, male = 1)	0.211 (0.267)	1.235 (0.731–2.085)	−0.259 (0.294)	0.772 (0.433–1.374)	−0.692 (0.498)	0.501 (0.189–1.328)
Age, years	0.035 (0.046)	1.035 (0.946–1.133)	0.056 (0.050)	1.058 (0.959–1.166)	0.154* (0.079)	1.167 (0.999–1.363)
Currently studying (0 = yes, 1 = no)	0.038 (0.425)	1.039 (0.452–2.389)	0.126 (0.425)	1.135 (0.493–2.611)	−1.218 (0.767)	0.296 (0.066–1.329)
Currently working (0 = yes, 1 = no)	0.086 (0.385)	1.090 (0.513–2.316)	−0.116 (0.397)	0.891 (0.409–1.939)	−0.286 (0.569)	0.751 (0.247–2.290)
Socio-economic status	0.248** (0.080)	1.281 (1.094–1.499)	0.069 (0.085)	1.071 (0.906–1.267)	−0.156 (0.141)	0.855 (0.649–1.127)
Caregiving context variables						
Number of family members	(0.140) 0.126	1.150 (0.899–1.471)	0.134 (0.135)	1.143 (0.878–1.489)	0.550** (0.171)	1.734 (1.239–2.426)
Ill parent (0 = mother; 1 = father)	−0.718 (0.588)	0.488 (0.154–1.544)	−0.139 (0.704)	0.870 (0.219–3.462)	−1.604* (0.735)	0.201 (0.048–0.849)
Parental illness (physical = 0, mental = 1)	−0.718 (0.406)	0.488 (0.220–1.082)	0.112 (0.372)	1.118 (0.540–2.316)	0.682 (0.540)	1.978 (0.686–5.697)
Both ill parents (0 = yes, 1 = no)	0.638 (0.547)	1.893 (0.648–5.531)	0.144 (0.647)	1.155 (0.325–4.108)	1.500* (0.670)	4.480 (1.205–16.657)
Amount of caregiving	−0.570** (0.169)	0.566 (0.406–0.788)	−0.233 (0.172)	0.792 (0.565–1.109)	−0.333 (0.268)	0.717 (0.424–1.212)

All latent profiles are compared to the ‘high BF & caregiving profile’ (*n* = 117). B = coefficient, SE = standard error. 95% CI = 95% confidence interval. ^∗^*p* < 0.05, **p < 0.01. Model χ^2^ (30) = 78.02, p < 0.001. Significant coefficients are displayed in bold.

Regarding caregiving context variables, the number of family members, ill parent’s gender, both ill parents, and amount of caregiving significantly influenced profile classification. Specifically, increased caregiving levels lowered the odds of being in the ‘low BF & caregiving profile’ compared to the ‘high BF & caregiving profile’ by 0.57 per increase in caregiving (B = −0.57, SE = 0.17, *p* < 0.01, 95% CI = 0.41–0.79). Each additional family member increased the odds of belonging to the ‘moderate BF & extremely high caregiving profile’ over the ‘high BF & caregiving profile’ by 1.73 times (B = 0.55, SE = 0.17, *p* < 0.01, 95% CI = 1.24–2.43). Having an ill father, versus an ill mother, reduced the likelihood of being classified in the ‘moderate BF & extremely high caregiving profile’ by a factor of 0.20 (B = 0.55, SE = 0.17, *p* < 0.01, 95% CI = 0.05–0.85), suggesting a higher probability for those with an ill mother to be classified in the ‘high BF & caregiving profile’. For participants with both parents ill, the odds of being in the ‘moderate BF & extremely high caregiving profile’ were 4.48 higher compared to the reference profile (B = 1.50, SE = 0.67, *p* < 0.05, 95% CI = 1.21–16.66).

### 3.4 Differences in HRQoL and mental health across profiles

To address objective 3, ANCOVAs explored differences in HRQoL and mental health across the four profiles after adjusting for age, SES, number of family members, ill parent’s gender, both ill parents, and amount of caregiving (see Table 5). Results showed significant effects of profile membership on HRQoL [*F* (3, 403) = 9.11, *p* < 0.01, Cohen’s d = 0.92], and mental health: internalizing [*F* (3, 403) = 5.92, *p* < 0.01, Cohen’s d = 0.74], and externalizing behaviors [*F* (3, 403) = 3.47, *p* < 0.01, Cohen’s d = 0.57], with effect sizes ranging from moderate-to-large for HRQoL and internalizing behaviors, and moderate for externalizing behaviors. The ‘low BF & caregiving profile’ exhibited the highest HRQoL and mental health, followed by the ‘high BF & caregiving profile’, with progressively lower HRQoL and mental health observed in the ‘moderate BF & high caregiving profile’. The lowest HRQoL and mental health emerged in the ‘moderate BF & extremely high caregiving profile’. *Post hoc* analyses showed that the ‘low BF & caregiving profile’ reported significantly better HRQoL and mental health compared to the two moderate BF profiles.

**Table 5:** Variations in HRQoL and mental health across the four profiles.

Variable	Total sample (*N* = 403)	Low BF & caregiving (*n* = 154)	High BF & caregiving (*n* = 117)	Moderate BF & high caregiving (*n* = 102)	Moderate BF & extremely high caregiving (*n* = 30)	Test of group difference	Cohen’s *d* for largest observed difference in pairs of profiles
	*M* (SD)	*M* (SD)	*M* (SD)	*M* (SD)	*M* (SD)		
HRQoL	95.06 (15.69)	99.74 (14.36)^a,c,d^	96.53 (14.37)^b,c,d^	89.46 (15.11)^a,b,c^	84.63 (18.82)^a,b,d^	*F* (3,403) = 9.11**	0.92
Mental health:							
Internalizing behaviors	15.79 (9.94)	12.42 (9.27)^a,b,c,d^	16.34 (8.63)^a,b,d^	18.17 (10.27)^a,c^	22.77 (10.91)^a,b,d^	*F* (3,403) = 5.92**	0.74
Externalizing behaviors	10.31 (6.843)	8.87 (6.25)^a,c,d^	10.91 (6.32)	11.22 (7.59)^a,c^	12.40 (7.71)^a,d^	*F* (3,403) = 3.47**	0.57

^∗^*p* < 0.01, **p < 0.001. Means sharing the same superscript notation (a,b,c,d) significantly differed in the post-hoc comparisons. Cohen’s d was derived from the eta-squared values obtained from the ANOVAs.

## 4 Discussion

This is the first study to use a person-oriented approach to explore patterns of variation in BF and caregiving in youth who have a parent with a serious physical or mental illness. The first study objective was to delineate profiles of BF and caregiving in youth caregivers in a parental illness context. LPAs revealed four profiles. Of these, the ‘low BF & caregiving profile’ (38.21%) had the highest proportion of participants, followed by the ‘high BF & caregiving profile’ (29.03%) and the ‘moderate BF & high caregiving profile’ (25.31%), with the ‘moderate BF & extremely high caregiving profile’ (7.45%) having the lowest proportion. This distribution reflects the caregiving continuum mentioned earlier. As might be expected, fewer youth caregivers were at the high end of the continuum, with the majority at the lower caregiving end. Similarly, the profiles suggest a continuum from low to high BF, indicating that engagement in BF among youth caregivers varies in accordance with the intensity of their caregiving.

Also reflecting the caregiving continuum are differences among profiles on specific caregiving dimensions. The ‘moderate BF & extremely high caregiving profile’ evidenced the highest caregiving impacts, particularly in global activity restrictions, activity restrictions in study/work, and caregiving stigma and resentment. This corresponds with research findings showing greater caregiving responsibilities are associated with more intense caregiving experiences (Landi et al., 2022c). Conversely, the ‘low BF & caregiving profile’ showed the lowest caregiving levels across all dimensions. Between these extremes were two profiles (‘high BF & caregiving’ and ‘moderate BF & high caregiving’ profiles) with relatively high caregiving. The greatest variations across the four profiles were on the caregiving dimensions of caregiving responsibilities, global activity restrictions, and study/work-related restrictions. These dimensions likely showed the greatest variations because they directly impact youth caregiver daily functioning and capacity to maintain personal and study/work commitments and are particularly affected by caregiving load (Pakenham et al., 2006).

Regarding BF, the ‘low BF & caregiving profile’ reported the lowest BF levels, while the ‘high BF & caregiving profile’ reported the highest BF levels. Corresponding levels of BF and caregiving covaried in these two profiles, with both BF and caregiving being either low or high. Such close correspondence was not apparent in the other two moderate BF profiles. The patterns of covariation between BF and caregiving characterizing each profile align with the proposal derived from BF theory that adversity must be sufficiently intense to disrupt meaning structures and thus trigger meaning restoration through a search for positives (Janoff-Bulman and Yopyk, 2004; Tedeschi and Calhoun, 2004). For example, the profile that reflects the low end of the caregiving continuum (‘low BF & caregiving’) is associated with correspondingly low BF because, according to BF theory, the adversity related to low caregiving engagement is unlikely to be intense enough to disrupt meaning and evoke BF. In contrast, the two profiles characterized by high caregiving exhibited correspondingly high-to-moderate BF. Higher caregiving levels are associated with greater negative impacts (Pakenham et al., 2006; Cox and Pakenham, 2014), and consistent with BF theory, such adversity is likely to trigger BF to restore meaning. However, adversity may be so intense that it overwhelms coping mechanisms like BF. This is reflected in the profile with the highest caregiving levels (‘moderate BF & extremely high caregiving’), where BF fails to reach the threshold at which it protects against the adverse impacts of very high caregiving.

Our second study objective was to explore associations between profile membership and socio-demographic and caregiving context variables. Regarding socio-demographics, SES emerged as a significant predictor; higher SES participants were more likely to be categorized in the ‘low BF & caregiving profile’ compared to the ‘high BF & caregiving profile’. This reflects previous research findings indicating that caregiving is more commonly undertaken by those from socio-economically disadvantaged backgrounds (Hunt et al., 2005; Nagl-Cupal et al., 2014; Metzing et al., 2020; Pilato et al., 2024; Landi et al., 2025a). Age also varied between profiles: compared to the ‘moderate BF & extremely high caregiving profile’, the ‘high BF & caregiving profile’ was more likely to include older youth, which aligns with prior research findings (Nagl-Cupal et al., 2014; Metzing et al., 2020).

Regarding caregiving context variables, caregiving load predicted profile membership: higher caregiving was linked to a lower likelihood of membership in the ‘low BF & caregiving profile’ compared to the ‘high BF & caregiving profile’, further validating the two ends of the caregiving continuum. Family size also predicted profile membership: a higher number of family members increased the likelihood of being classified in the ‘moderate BF & extremely high caregiving profile’ rather than the ‘high BF & caregiving profile’. It is possible that having more family members increases the caregiving load (Nagl-Cupal et al., 2014; Pilato et al., 2024). Additionally, the gender of the ill parent predicted profile membership. Specifically, youth with an ill father were less likely to be classified in the ‘moderate BF & extremely high caregiving profile’ compared to the ‘high BF & caregiving profile’. Research indicates higher caregiving in youth with an ill mother (Joseph et al., 2019; Leu et al., 2019; Metzing et al., 2020), possibly because mothers assume a greater share of household tasks. Lastly, the presence of both ill parents significantly increased the likelihood of being in the ‘moderate BF & extremely high caregiving profile’ rather than the ‘high BF & caregiving profile’, highlighting the profound impact of living with both ill parents on youth caregiving.

Our final study objective was to investigate differences across profiles in HRQoL and mental health (internalizing and externalizing behaviors) after controlling for the effects of socio-demographics and caregiving context variables that profiles differed on. Results showed that HRQoL and mental health varied along the caregiving continuum: the ‘low BF & caregiving profile’, at the lowest end, showed the highest HRQoL and mental health, whereas the ‘moderate BF & extremely high caregiving profile’, at the very highest end, evidenced the lowest HRQoL and mental health. These results are consistent with prior research showing that higher caregiving predicts poorer mental health (Pakenham and Cox, 2015; Landi et al., 2022c). Between the two ends of the continuum were the two profiles with relatively high caregiving and varying levels of BF: ‘high BF & caregiving profile’ and ‘moderate BF & high caregiving profile.’ Notably, the ‘high BF & caregiving profile’ had the second highest levels of HRQoL and mental health, while the ‘moderate BF & high caregiving profile’ had the third highest. The interplay between BF and caregiving in these two profiles demonstrates the protective role of BF. That is, despite high caregiving levels, these profiles maintained moderately high levels of HRQoL and mental health. These results suggest BF buffers against the adverse impacts of high caregiving. However, it appears that to be protective, engagement in BF must be elevated to a critical threshold relative to increasing caregiving levels. This is evident in the profile with the poorest HRQoL and mental health, which also reported only moderate BF levels but extremely high caregiving (‘moderate BF & extremely high caregiving profile’). In the context of extremely high caregiving, correspondingly high BF levels are likely necessary to mitigate the negative impacts of very high caregiving. Overall, these findings align with previous research showing that BF moderates the inverse association between caregiving and mental health among caregivers aged 15–21 caring for an ill family member (Wepf et al., 2022), and that higher BF predicts better mental health and ameliorates the negative impacts of caregiving on adjustment in youth aged 9–20 caring for an ill parent (Pakenham and Cox, 2018). Our study expands on this by identifying patterns of engagement in caregiving and BF in youth caregivers aged 11–24.

Despite a trend suggesting lower externalizing behaviors in the ‘moderate BF & high caregiving’ and ‘high BF & caregiving’ profiles compared to the ‘moderate BF & extremely high caregiving profile’, *post-hoc* comparisons showed these differences were nonsignificant. This implies that BF does not shield against the adverse effects of caregiving on externalizing behaviors, suggesting that different mechanisms may be at play. Caregiving benefit finding could be more relevant to internalizing behaviors because of the associated introspection (McElroy, 2009).

Regarding practice implications, results suggest that youth in a parental illness context who engage in very high levels of caregiving that overwhelm coping mechanisms, such as BF, are at risk of significant deficits in HRQoL and poor mental health and should be targeted with support services, particularly those that reduce youth caregiving responsibilities. Relevant sectors (e.g., education, health, employment, and training) should be sensitised to the needs of youth living in a parental illness context. In particular, helping professionals—such as clinical and school psychologists—can play a key role in assessment and intervention. Regarding assessment, routine screening of caregiving load and emotional distress should be implemented, and where indicated, followed by referral to appropriate support services or psychosocial intervention provided by the helping professional where they are suitably qualified. Interventions could include psychoeducation about caregiving stress, emotional regulation skills, and strategies for reframing caregiving experiences as personally meaningful and enriching. Regarding the latter, in view of the results supporting the protective role of BF, youth caregivers who engage in relatively high caregiving should be encouraged to explore the positive aspects of their caregiving role. BF interventions for youth caregivers could be established, as has been developed for adult caregivers (Cheng et al., 2017). These may include guided reflection, strengths-based strategies, and cognitive-behavioral techniques aimed at enhancing meaning-making in youth caregiving. However, facilitation of BF must recognise the costs and distress associated with caring for an ill parent (Tedeschi and Calhoun, 2004). Indeed, research shows that wellbeing is maximised when there is a balance between realistic perceptions of both the positives and negatives in adversity (Cheng et al., 2006).

### 4.1 Study limitations and future research

This study has several methodological limitations. The non-random sampling limits the generalizability of findings, and the cross-sectional study design prevents the establishment of causal links among study variables. Furthermore, while the wide age range of participants is potentially problematic, only two profiles differed in age, and age was controlled for in analyses investigating differences among profiles in HRQoL and mental health. Information on parental illness severity, prognosis, and duration was not assessed and may influence youth caregiver BF. Longitudinal studies are necessary and should include young children, providing insights into the dynamics of BF and caregiving over time. Future research should also evaluate the effects of BF interventions on HRQoL and mental health in youth caregivers.

## 5 Conclusion

In line with the first study objective, the person-oriented approach used in this research identified four empirically distinct profiles of BF and caregiving among youth caring for a parent with a serious physical or mental illness. The distribution of caregiving and participants across profiles mirrored the caregiving continuum. The lowest caregiving and highest proportion of participants marked the continuum’s low end, while the highest caregiving and lowest proportion of participants marked the high end. The remaining two profiles fell between these two poles, reflecting mid-range caregiving and participant proportions. Regarding the second study objective, the four profiles differed in socio-demographics and caregiving contextual factors—including socio-economic status, age, family size, and parental illness characteristics. Regarding the third study objective, after controlling for relevant socio-demographic and caregiving context variables, the mid-caregiving continuum profiles reported high-to-moderate BF and demonstrated better HRQoL and mental health than the profile with the highest caregiving. Hence, these two mid-continuum profiles illustrated the protective role of BF, as despite high caregiving, these profiles evidenced moderately high HRQoL and mental health. This study suggests that BF buffers against the adverse impacts of high caregiving. Findings also supported the BF theoretical proposal that caregiving must be sufficiently intense to trigger BF. Additionally, findings suggest that youth in a parental illness context who engage in very high caregiving levels are at risk of poor HRQoL and mental health. Support services should reduce youth caregiving responsibilities and encourage youth caregivers to explore the positive aspects of their caregiving roles.

## Data Availability

The raw data supporting the conclusions of this article will be made available by the authors, without undue reservation.

## References

[ref1] Achenbach T. M. Rescorla L. A. (2001). Manual for the ASEBA school-age forms and profiles. Burlington (VT): University of Vermont, Research Center for Children, Youth, & Families.

[ref2] Areguy F. Mock S. E. Breen A. Van Rhijn T. Wilson K. Lero D. S. (2019). Communal orientation, benefit-finding, and coping among young carers. Child Youth Serv. 40, 363–382. doi: 10.1080/0145935X.2019.1614906

[ref3] Becker S. (2007). Global perspectives on children’s unpaid caregiving in the family: research and policy on ‘young carers’ in the UK, Australia, the USA and sub-Saharan Africa. Glob. Soc. Policy 7, 23–50. doi: 10.1177/1468018107073892

[ref4] Bergman L. R. Andersson H. (2010). The person and the variable in developmental psychology. J. Psychol. 218, 155–165. doi: 10.1027/0044-3409/a000025

[ref5] Berlin K. S. Williams N. A. Parra G. R. (2014). An introduction to latent variable mixture modeling (part 1): overview and cross-sectional latent class and latent profile analyses. J. Pediatr. Psychol. 39, 174–187. doi: 10.1093/jpepsy/jst084, PMID: 24277769

[ref6] Bolas H. Wersch A. V. Flynn D. (2007). The well-being of young people who care for a dependent relative: an interpretative phenomenological analysis. Psychol. Health 22, 829–850. doi: 10.1080/14768320601020154

[ref7] Bollen K. A. (1989). Structural equations with latent variables. Chapel Hill, North Carolina: John Wiley & Sons.

[ref8] Bonadio F. T. Evans S. C. Cho G. Y. Callahan K. P. Chorpita B. F. Weisz J. R.. (2022). Whose outcomes come out? Patterns of caregiver- and youth-reported outcomes based on caregiver-youth baseline discrepancies. J. Clin. Child Adolesc. Psychol. 51, 469–483. doi: 10.1080/15374416.2021.1955367, PMID: 34424107

[ref9] Boyce W. Torsheim T. Currie C. Zambon A. (2006). The family affluence scale as a measure of national wealth: validation of an adolescent self-report measure. Soc. Indic. Res. 78, 473–487. doi: 10.1007/s11205-005-1607-6

[ref10] Cassidy T. (2013). Benefit finding through caring: the cancer caregiver experience. Psychol. Health 28, 250–266. doi: 10.1080/08870446.2012.717623 22928621

[ref11] Cassidy T. Giles M. (2013). Further exploration of the young Carers perceived stress scale: identifying a benefit-finding dimension. Br. J. Health Psychol. 18, 642–655. doi: 10.1111/bjhp.12017, PMID: 23279322

[ref12] Cheng S.-T. Mak E. P. M. Fung H. H. Kwok T. Lee D. T. F. Lam L. C. W. (2017). Benefit-finding and effect on caregiver depression: a double-blind randomized controlled trial. J. Consult. Clin. Psychol. 85, 521–529. doi: 10.1037/ccp0000176, PMID: 28287803

[ref13] Cheng C. Wong W. Tsang K. W. (2006). Perception of benefits and costs during SARS outbreak: an 18-month prospective study. J. Consult. Clin. Psychol. 74, 870–879. doi: 10.1037/0022-006X.74.5.870, PMID: 17032091

[ref14] Cohn L. N. Pechlivanoglou P. Lee Y. Mahant S. Orkin J. Marson A.. (2020). Health outcomes of parents of children with chronic illness: a systematic review and meta-analysis. J. Pediatr. 218, 166–177.e2. doi: 10.1016/j.jpeds.2019.10.068, PMID: 31916997

[ref15] Cox S. D. Pakenham K. I. (2014). Confirmatory factor analysis and invariance testing of the young Carer of parents inventory (YCOPI). Rehabil. Psychol. 59, 439–452. doi: 10.1037/a0035860, PMID: 25150807

[ref16] Earley L. Cushway D. Cassidy T. (2007). Children’s perceptions and experiences of care giving: a focus group study. Couns. Psychol. Q. 20, 69–80. doi: 10.1080/09515070701217830

[ref17] Ellis P. D. (2010). The essential guide to effect sizes: statistical power, meta-analysis, and the interpretation of research results. New York, USA: Cambridge University Press.

[ref18] Frigerio A. Cattaneo C. Cataldo M. Schiatti A. Molteni M. Battaglia M. (2004). Behavioral and emotional problems among italian children and adolescents aged 4 to 18 years as reported by parents and teachers. Eur. J. Psychol. Assess. 20, 124–133. doi: 10.1027/1015-5759.20.2.124

[ref19] Gough G. Gulliford A. (2020). Resilience amongst young carers: investigating protective factors and benefit-finding as perceived by young carers. Educ. Psychol. Pract. 36, 149–169. doi: 10.1080/02667363.2019.1710469

[ref20] Hendricks B. A. Vo J. B. Dionne-Odom J. N. Bakitas M. A. (2021). Parentification among young carers: a concept analysis. Child Adolesc. Soc. Work J. 38, 519–531. doi: 10.1007/s10560-021-00784-7, PMID: 38828384 PMC11142575

[ref21] Hunt G. Levine C. Naiditch L. (2005). Young caregivers in the US: Findings from a national survey. Bethesda: National Alliance for Caregiving.

[ref22] Jamir Singh P. S. Azman A. Drani S. Mohd Nor M. I. H. Che Ahmad A. (2023). Navigating the terrain of caregiving of children with intellectual and developmental disabilities: importance of benefit finding and optimism. Humanit. Soc. Sci. Commun. 10, 1–7. doi: 10.1057/s41599-023-02211-x

[ref23] Janoff-Bulman R. Yopyk D. J. (2004). “Random outcomes and valued commitments: existential dilemmas and the paradox of meaning” in Handbook of experimental existential psychology. eds. Greenberg J. Koole S. L. Pyszczynski T. (New York, NY: The Guilford Press), 122–138.

[ref24] Joseph S. Kendall C. Toher D. Sempik J. Holland J. Becker S. (2019). Young carers in England: findings from the 2018 BBC survey on the prevalence and nature of caring among young people. Child Care Health Dev. 45, 606–612. doi: 10.1111/cch.12674, PMID: 30995694

[ref25] Joseph S. Sempik J. Leu A. Becker S. (2020). Young carers research, practice and policy: an overview and critical perspective on possible future directions. Adolesc. Res. Rev. 5, 77–89. doi: 10.1007/s40894-019-00119-9

[ref26] Kircanski K. Zhang S. Stringaris A. Wiggins J. L. Towbin K. E. Pine D. S.. (2017). Empirically derived patterns of psychiatric symptoms in youth: a latent profile analysis. J. Affect. Disord. 216, 109–116. doi: 10.1016/j.jad.2016.09.016, PMID: 27692699 PMC5360533

[ref27] Kritikos T. K. Stiles-Shields C. Winning A. M. Starnes M. Ohanian D. M. Clark O. E.. (2021). A systematic review of benefit-finding and growth in pediatric medical populations. J. Pediatr. Psychol. 46, 1076–1090. doi: 10.1093/jpepsy/jsab041, PMID: 34382081 PMC8628652

[ref28] Lacey R. E. Xue B. McMunn A. (2022). The mental and physical health of young carers: a systematic review. Lancet Public Health 7, e787–e796. doi: 10.1016/S2468-2667(22)00161-X, PMID: 36057277

[ref29] Landi G. Boccolini G. Giovagnoli S. Pakenham K. I. Grandi S. Tossani E. (2022a). Validation of the Italian young Carer of parents inventory-revised (YCOPI-R). Disabil. Rehabil. 44, 795–806. doi: 10.1080/09638288.2020.1780478, PMID: 32567411

[ref30] Landi G. Grangel A. B. Pakenham K. I. Gallagher S. Grandi S. Tossani E. (2025a). Adverse childhood experiences (ACEs) in young adult carers relative to non-carer peers and relations with mental health, caregiving and socio-demographics. Child Indic. Res. doi: 10.1007/s12187-025-10237-7

[ref31] Landi G. Pakenham K. I. Bao Z. Cattivelli R. Crocetti E. Tossani E.. (2025b). Efficacy of psychosocial interventions for young offspring of parents with a serious physical or mental illness: systematic review and meta-analysis. Clin. Psychol. Rev. 118:102569. doi: 10.1016/j.cpr.2025.102569, PMID: 40179592

[ref32] Landi G. Pakenham K. I. Cattivelli R. Grandi S. Tossani E. (2022b). Caregiving responsibilities and mental health outcomes in young adult carers during the covid-19 pandemic: a longitudinal study. Int. J. Environ. Res. Public Health 19:15149. doi: 10.3390/ijerph192215149, PMID: 36429866 PMC9690746

[ref33] Landi G. Pakenham K. I. Crocetti E. Grandi S. Tossani E. (2022c). Examination of the tripartite model of youth caregiving in the context of parental illness. Psychol. Health 37, 397–418. doi: 10.1080/08870446.2020.1870116, PMID: 33417502

[ref34] Landi G. Pakenham K. I. Grandi S. Tossani E. (2022d). Young adult carers during the pandemic: the effects of parental illness and other ill family members on covid-19-related and general mental health outcomes. Int. J. Environ. Res. Public Health 19:3391. doi: 10.3390/ijerph19063391, PMID: 35329079 PMC8950288

[ref35] Lanza S. T. Cooper B. R. (2016). Latent class analysis for developmental research. Child Dev. Perspect. 10, 59–64. doi: 10.1111/cdep.12163, PMID: 31844424 PMC6914261

[ref36] Leu A. Frech M. Wepf H. Sempik J. Joseph S. Helbling L.. (2019). Counting young carers in Switzerland - a study of prevalence. Child. Soc. 33, 53–67. doi: 10.1111/chso.12296

[ref37] Little R. J. A. (1988). A test of missing completely at random for multivariate data with missing values. J. Am. Stat. Assoc. 83, 1198–1202. doi: 10.1080/01621459.1988.10478722

[ref38] McDonald J. Cumming J. Dew K. (2009). An exploratory study of young carers and their families in New Zealand. Kotuitui 4, 115–129. doi: 10.1080/1177083X.2009.9522448, PMID: 40101104

[ref39] McDougall E. O’Connor M. Howell J. (2018). “Something that happens at home and stays at home”: an exploration of the lived experience of young carers in Western Australia. Health Soc. Care Community 26, 572–580. doi: 10.1111/hsc.12547, PMID: 29457295

[ref40] McElroy S. K. (2009). The role of meaning making in the association between multiple interpersonal traumas and post -traumatic adaptation. [Doctoral dissertation], Bowling Green State University. Available online at. https://www.proquest.com/openview/d24221e8771bf3ebe5d85c8bbb342c29/1?pq-origsite=gscholar&cbl=18750 (Accessed April 19, 2024).

[ref41] McGorry P. D. Mei C. Dalal N. Alvarez-Jimenez M. Blakemore S.-J. Browne V.. (2024). The lancet psychiatry commission on youth mental health. Lancet Psychiatry 11, 731–774. doi: 10.1016/S2215-0366(24)00163-9, PMID: 39147461

[ref42] Metzing S. Ostermann T. Robens S. Galatsch M. (2020). The prevalence of young carers – a standardised survey amongst school students (KiFam-study). Scand. J. Caring Sci. 34, 501–513. doi: 10.1111/scs.12754, PMID: 31657036

[ref43] Metzing-Blau S. Schnepp W. (2008). Young carers in Germany: to live on as normal as possible – a grounded theory study. BMC Nurs. 7:15. doi: 10.1186/1472-6955-7-15, PMID: 19108719 PMC2627850

[ref44] Muthén L. K. Muthén B. O. (2018). Mplus User’s Guide. 8th Edn.

[ref45] Nagl-Cupal M. Daniel M. Koller M. M. Mayer H. (2014). Prevalence and effects of caregiving on children. J. Adv. Nurs. 70, 2314–2325. doi: 10.1111/jan.12388, PMID: 24660847

[ref46] Newman T. (2002). “Young Carers” and disabled parents: time for a change of direction? Disabil. Soc. 17, 613–625. doi: 10.1080/0968759022000010407

[ref47] Nichols K. R. Fam D. Cook C. Pearce M. Elliot G. Baago S.. (2013). When dementia is in the house: needs assessment survey for young caregivers. Can. J. Neurol. Sci. 40, 21–28. doi: 10.1017/S0317167100012907, PMID: 23250123

[ref48] Nylund-Gibson K. Choi A. Y. (2018). Ten frequently asked questions about latent class analysis. Transl. Iss. Psychol. Sci. 4, 440–461. doi: 10.1037/tps0000176

[ref49] Pakenham K. I. (2005). The positive impact of multiple sclerosis (MS) on carers: associations between carer benefit finding and positive and negative adjustment domains. Disabil. Rehabil. 27, 985–997. doi: 10.1080/09638280500052583, PMID: 16096252

[ref50] Pakenham K. I. Bursnall S. (2006). Relations between social support, appraisal and coping and both positive and negative outcomes for children of a parent with multiple sclerosis and comparisons with children of healthy parents. Clin. Rehabil. 20, 709–723. doi: 10.1191/0269215506cre976oa, PMID: 16944828

[ref51] Pakenham K. I. Bursnall S. Chiu J. Cannon T. Okochi M. (2006). The psychosocial impact of caregiving on young people who have a parent with an illness or disability: comparisons between young caregivers and noncaregivers. Rehabil. Psychol. 51, 113–126. doi: 10.1037/0090-5550.51.2.113

[ref52] Pakenham K. I. Chiu J. Bursnall S. Cannon T. (2007). Relations between social support, appraisal and coping and both positive and negative outcomes in young carers. J. Health Psychol. 12, 89–102. doi: 10.1177/1359105307071743, PMID: 17158843

[ref53] Pakenham K. I. Cox S. (2008). Development of the benefit finding in multiple sclerosis (MS) caregiving scale: a longitudinal study of relations between benefit finding and adjustment. Br. J. Health Psychol. 13, 583–602. doi: 10.1348/135910707X250848 18854061

[ref54] Pakenham K. I. Cox S. (2012). Test of a model of the effects of parental illness on youth and family functioning. Health Psychol. 31, 580–590. doi: 10.1037/a0026530, PMID: 22149127

[ref55] Pakenham K. I. Cox S. (2015). The effects of parental illness and other ill family members on youth caregiving experiences. Psychol. Health 30, 857–878. doi: 10.1080/08870446.2014.1001390, PMID: 25565077

[ref56] Pakenham K. I. Cox S. (2018). Effects of benefit finding, social support and caregiving on youth adjustment in a parental illness context. J. Child Fam. Stud. 27, 2491–2506. doi: 10.1007/s10826-018-1088-2

[ref57] Park C. L. Folkman S. (1997). Meaning in the context of stress and coping. Rev. Gen. Psychol. 1, 115–144. doi: 10.1037/1089-2680.1.2.115

[ref58] Pedersen S. Revenson T. A. (2005). Parental illness, family functioning, and adolescent well-being: a family ecology framework to guide research. J. Fam. Psychol. 19, 404–419. doi: 10.1037/0893-3200.19.3.404, PMID: 16221021

[ref59] Pilato J. Dorard G. Untas A. (2024). Prevalence and quality of life of 11–15-year-old adolescent young carers in France: a school-based study. Eur. Child Adolesc. Psychiatry 33, 3101–3110. doi: 10.1007/s00787-024-02383-0, PMID: 38353678

[ref60] Ravens-Sieberer U. Auquier P. Erhart M. Gosch A. Rajmil L. Bruil J.. (2007). The KIDSCREEN-27 quality of life measure for children and adolescents: psychometric results from a cross-cultural survey in 13 European countries. Qual. Life Res. 16, 1347–1356. doi: 10.1007/s11136-007-9240-2, PMID: 17668292

[ref61] Sieh D. S. Meijer A. M. Oort F. J. Visser-Meily J. M. A. Van der Leij D. A. V. (2010). Problem behavior in children of chronically ill parents: a meta-analysis. Clin. Child. Fam. Psychol. Rev. 13, 384–397. doi: 10.1007/s10567-010-0074-z, PMID: 20640510 PMC2975921

[ref62] Taylor S. E. (1983). Adjustment to threatening events: a theory of cognitive adaptation. Am. Psychol. 38, 1161–1173. doi: 10.1037/0003-066X.38.11.1161

[ref63] Tedeschi R. G. Calhoun L. G. (2004). Posttraumatic growth: conceptual foundations and empirical evidence. Psychol. Inq. 15, 1–18. doi: 10.1207/s15327965pli1501_01

[ref64] Tennen H. Affleck G. (2002). “Benefit-finding and benefit-reminding” in Handbook of positive psychology. eds. Snyder C. R. Lopez S. J. (New York, NY, USA: Oxford University Press), 584–597.

[ref65] Untas A. Jarrige E. Vioulac C. Dorard G. (2022). Prevalence and characteristics of adolescent young carers in France: the challenge of identification. J. Adv. Nurs. 78, 2367–2382. doi: 10.1111/jan.15162, PMID: 35112732

[ref66] van Widenfelt B. M. Treffers P. D. A. de Beurs E. Siebelink B. M. Koudijs E. (2005). Translation and cross-cultural adaptation of assessment instruments used in psychological research with children and families. Clin. Child. Fam. Psychol. Rev. 8, 135–147. doi: 10.1007/s10567-005-4752-1, PMID: 15981582

[ref67] von Rezori R. E. Baumeister H. Holl R. W. Minden K. Müller-Stierlin A. S. Reinauer C.. (2024). Testing a model of benefit-finding and growth in youths with chronic health conditions. BMC Pediatr. 24:19. doi: 10.1186/s12887-023-04467-3, PMID: 38183031 PMC10768283

[ref68] Wepf H. Joseph S. Leu A. (2022). Benefit finding moderates the relationship between young carer experiences and mental well-being. Psychol. Health 37, 1270–1286. doi: 10.1080/08870446.2021.1941961, PMID: 34180332

